# Supplementary material for: Activation of the CaMKII-Sarm1-ASK1-p38 MAP kinase pathway protects against axon degeneration caused by loss of mitochondria
Source: eLife. 2022 Mar 14;11:e73557. doi: 10.7554/eLife.73557 (PMC8920508; doi:10.7554/eLife.73557)
Supplement: Figure 5—figure supplement 1—source data 1. [file elife-73557-fig5-figsupp1-data1.zip › Figure 5-figure supplement 1-source data1/figure 5-figure supplement 1-source data1-legends.pdf]

Figure 5-figure supplement 1-source data 1 legends

Figure 5-figure supplement 1-source data 1. Gel images of APL-1 genotyping
Three extrachromosomal array lines (*oyIs14[sra-6p::GFP], ric-7(n2657) V; apl-1(wp19) X + sra-6p::nCre)* were generated from one microinjection and genotyped for APL KO in PVQ. 15 worms from line 1, 8 worms from line 2 and 8 worms from line 3 were genotyped. The KO band(583bp) can be seen in most worms from line 1 but not in worms from line 2 or line 3, probably due to expression levels and mosaicism. Line 1 was then integrated to generate *XE2634(oyIs14[sra-6p::GFP], ric-7(n2657) V; apl-1(wp19) X; wpIs146[sra-6p::nCre + odr-1p::RFP])*, which was used for analysis of axon degeneration. Control is *XE2415(oyIs14[sra-6p::GFP], ric-7(n2657) V; apl-1(wp19) X)*. Blank is no worm input. The cropped image of control and last three worms from line 1 is shown in figure 5-figure supplement 1B, with enhanced contrast.
