# Supplementary figures and images for: Activation of the CaMKII-Sarm1-ASK1-p38 MAP kinase pathway protects against axon degeneration caused by loss of mitochondria
Source: eLife. 2022 Mar 14;11:e73557. doi: 10.7554/eLife.73557 (PMC8920508; doi:10.7554/eLife.73557)

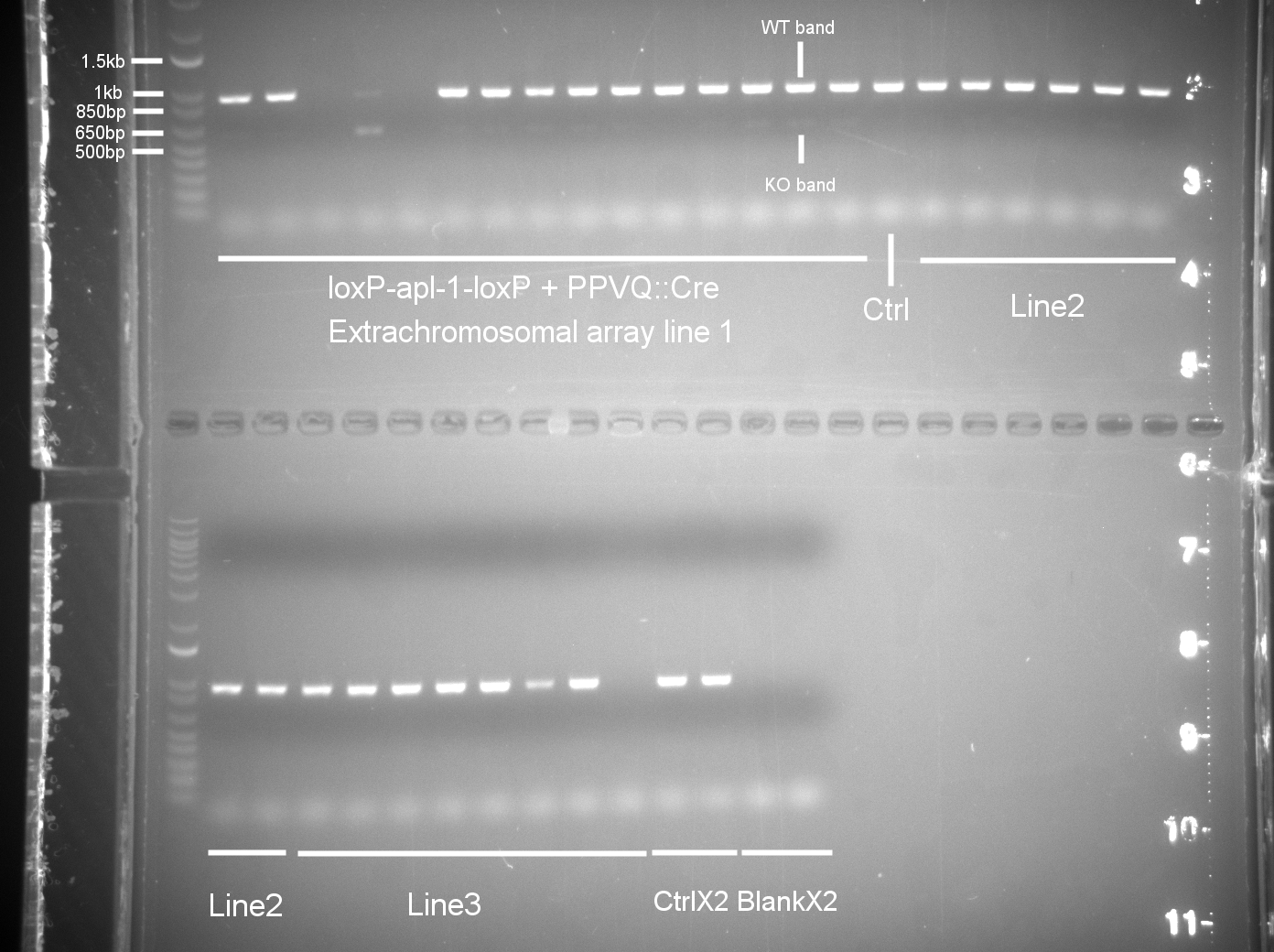

Supplement: Figure 5—figure supplement 1—source data 1. [file elife-73557-fig5-figsupp1-data1.zip › Figure 5-figure supplement 1-source data1/Figure 5-figure supplement 1-source data1 labelled.1sc.tif]
